# Belowground Inoculation With Arbuscular Mycorrhizal Fungi Increases Local and Systemic Susceptibility of Rice Plants to Different Pest Organisms

**DOI:** 10.3389/fpls.2018.00747

**Published:** 2018-06-05

**Authors:** Lina Bernaola, Marco Cosme, Raymond W. Schneider, Michael Stout

**Affiliations:** ^1^Department of Entomology, Louisiana State University Agricultural Center, Baton Rouge, LA, United States; ^2^Laboratory of Mycology, Earth and Life Institute, Université catholique de Louvain, Louvain-la-Neuve, Belgium; ^3^Department of Plant Pathology and Crop Physiology, Louisiana State University Agricultural Center, Baton Rouge, LA, United States

**Keywords:** arbuscular mycorrhizal fungi, rice, root colonization, rice water weevil, fall armyworm, sheath blight, aboveground-belowground interactions

## Abstract

Plants face numerous challenges from both aboveground and belowground stressors, and defend themselves against harmful insects and microorganisms in many ways. Because plant responses to biotic stresses are not only local but also systemic, belowground interactions can influence aboveground interactions in both natural and agricultural ecosystems. Arbuscular mycorrhizal fungi (AMF) are soilborne organisms that form symbiotic associations with many plant roots and are thought to play a central role in plant nutrition, growth, and fitness. In the present study, we focused on the influence of AMF on rice defense against pests. We inoculated rice plants with AMF in several field and greenhouse experiments to test whether the interaction of AMF with rice roots changes the resistance of rice against two chewing insects, the rice water weevil (*Lissorhoptrus oryzophilus* Kuschel, RWW) and the fall armyworm (*Spodoptera frugiperda*, FAW), and against infection by sheath blight (*Rhizoctonia solani*, ShB). Both in field and greenhouse experiments, the performance of insects and the pathogen on rice was enhanced when plants were inoculated with AMF. In the field, inoculating rice plants with AMF resulted in higher numbers of RWW larvae on rice roots. In the greenhouse, more RWW first instars emerged from AMF-colonized rice plants than from non-colonized control plants. Weight gains of FAW larvae were higher on rice plants treated with AMF inoculum. Lesion lengths and susceptibility to ShB infection were higher in rice plants colonized by AMF. Although AMF inoculation enhanced the growth of rice plants, the nutritional analyses of root and shoot tissues indicated no major increases in the concentrations of nutrients in rice plants colonized by AMF. The large effects on rice susceptibility to pests in the absence of large effects on plant nutrition suggest that AMF colonization influences other mechanisms of susceptibility (e.g., defense signaling processes). This study represents the first study conducted in the U.S. in rice showing AMF-induced plant susceptibility to several antagonists that specialize on different plant tissues. Given the widespread occurrence of AMF, our findings will help to provide a different perspective into the causal basis of rice systemic resistance/susceptibility to insects and pathogens.

## Introduction

Plants are active organisms capable of adapting to fluctuating environmental conditions; accordingly, they exhibit a high degree of phenotypic plasticity (Pozo et al., 2015). As an important example, plants respond to diverse biotic threats from above- and belowground herbivores and pathogens using a variety of direct and indirect defense mechanisms (Kessler and Baldwin, 2002; Robert-Seilaniantz et al., 2011). Because plant responses to herbivores and pathogens are both local and systemic, above- and belowground organisms may influence each other’s fitness through changes in the shared host plant (Bezemer and van Dam, 2005; Soler et al., 2007; Soler et al., 2009; Ali et al., 2013). The presence of soilborne microbes in the rhizosphere plays a considerable role in ecosystem functioning by changing nutrient uptake by plants (thereby influencing quality of the host plant for herbivores), promoting plant growth, and altering plant defense pathways independently of plant nutrition (van der Heijden et al., 1998; Pozo and Azcon-Aguilar, 2007; Smith and Read, 2008). The interplay of these various changes controls the final impact of soilborne microbes on the structure of communities associated with plants.

Arbuscular mycorrhizal fungi (AMF) are well-known, essential components of soil biota within natural and agricultural ecosystems (Smith and Read, 2008). AMF form associations with the root systems of more than 85% of vascular plant species, including many important crops (Smith and Read, 2008). The symbiosis between AMF and plants results in a continuum of effects on plant growth and fitness, from highly mutualistic to antagonistic (Johnson et al., 1997; Smith and Read, 2008; Currie et al., 2011; Barber et al., 2013b). Most often, however, associations with AMF facilitate the acquisition by plants of essential nutrients such as nitrogen, phosphate, and water from the soil (Smith and Read, 2008). In exchange, the fungal partner receives photosynthetically fixed carbon, which is used to grow more mycelial networks that allow the root system to expand in the soil and absorb more nutrients (Parniske, 2008; Smith and Read, 2008; Bonfante and Genre, 2010). Although in agricultural ecosystems the association of plants with AMF often results in plant yield increases (Gosling et al., 2006), the effects of AMF can also vary markedly along a parasitism-mutualism continuum (Johnson et al., 1997; Paszkowski, 2006; Fesel and Zuccaro, 2016). Because AMF are important components of soil microbial communities and are a central part of agro-ecosystems, they can potentially provide benefits but also costs to farmers.

Colonization of plant roots by AMF has been shown to alter plant quality for both above- and belowground insect herbivores and pathogens (Goverde et al., 2000; Gange, 2001; Koricheva et al., 2009; Currie et al., 2011) and AMF can contribute to improved resistance or tolerance against abiotic (Ruiz-Sanchez et al., 2010; Maya and Matsubara, 2013) and biotic stresses, such as those caused by root and shoot herbivores and pathogens (Gange, 2001; Pozo and Azcon-Aguilar, 2007; Smith and Read, 2008; Campos-Soriano et al., 2011; Vannette and Hunter, 2011). However, the effects of mycorrhizal colonization on insect fitness or pathogen infection vary depending on the identity of both AMF and host plant, the insect or pathogen involved, and environmental factors (Gange and West, 1994; Gange, 2001, 2007; Gange et al., 2002; Bennett et al., 2006; Borowicz, 2009; Gehring and Bennett, 2009; Koricheva et al., 2009; Pineda et al., 2010; Campos-Soriano et al., 2011; Currie et al., 2011; Vannette and Hunter, 2011). It has been proposed that generalist herbivores and necrotrophic pathogens are usually negatively affected by the presence of AMF, whereas specialist herbivores and biotrophic pathogens are usually positively affected, performing better on mycorrhizal plants (Gange et al., 2002; Hartley and Gange, 2009; Koricheva et al., 2009; Currie et al., 2011; Borowicz, 2013). A meta-analysis of 34 studies showed that AMF predominantly have negative effects on the performance of generalist chewing herbivores, but positive effects on specialist chewing insects (Koricheva et al., 2009).

The mechanisms by which mycorrhizal colonization alters plant resistance, and the effects of agricultural practices on the presence and effectiveness of AMF symbiosis in crop plants, are not fully understood. Increases in plant growth and improvements in nutrient uptake resulting from mycorrhizal colonization might make plants more attractive or susceptible to herbivores and pathogens (Roger et al., 2013). Alternatively, evidence from tomato plants showed that mycorrhizal colonization may change plant resistance by altering plant defense such as the jasmonic acid pathways (Jung et al., 2012). A large body of evidence also shows that insect herbivores and plant pathogens frequently induce plant defense responses, but the indirect effects of AMF on these induced responses are not thoroughly understood. Importantly, agricultural practices often reduce the presence and effectiveness of AMF symbiosis in the soil (Barber et al., 2013b), which may reduce or delay colonization of the crop by AMF relative to herbivore infestation or pathogen attack. A better understanding of the changes in crop plants in response to root colonization by AMF in agricultural settings, principally in major crops, and how these changes affect plant-herbivore or plant-pathogen relationships, is urgently needed to more effectively utilize mycorrhizae in agriculture.

Cereal crops are an important group of plants that establish symbiotic associations with AMF (Sawers et al., 2008; Gutjahr et al., 2009; Vallino et al., 2009; Campos-Soriano et al., 2011; Gutjahr et al., 2015b). Rice (*Oryza sativa* L) is a staple for more than half the globe’s population and represents a promising model system for studies of AMF interactions in general and plant-AMF-herbivore interactions in particular. The presence of AMF associations in rice roots has received increased attention in recent years (Gutjahr et al., 2009; Campos-Soriano et al., 2011; Edwards et al., 2015). In a recent study, a detailed characterization of the root-associated microbiomes of the rice plant revealed dynamic changes in these microbial communities as a function of geographical location, soil source, host genotype, and cultivation practices (Edwards et al., 2015). However, only a few studies have investigated the interacting effects of AMF symbiosis in rice plants and the implications of these interactions for insect herbivores or pathogens (Campos-Soriano et al., 2011; Cosme et al., 2011). For instance, mycorrhizal rice plants showed enhanced resistance to the rice blast fungus, *Magnaporthe oryzae* and this resistance appeared to rely on both the systemic activation of defense regulatory genes in the absence of pathogen challenge and priming for stronger expression of defense genes during pathogen infection (Campos-Soriano et al., 2011).

The aim of the current study was to understand how AMF inoculation influences rice-herbivore and rice-pathogen interactions. We used as model organisms three important pests of rice in the southern U.S.: larvae of the rice water weevil (RWW; *Lissorhoptrus oryzophilus* Kuschel; Coleoptera: Curculionidae), larvae of the fall armyworm (FAW, *Spodoptera frugiperda* J.E. Smith; Lepidoptera: Noctuidae), and sclerotia of sheath blight (ShB, *Rhizoctonia solani;* Basidiomycete). Of these three study organisms, only the effects of AMF on rice water weevils have been previously investigated. Cosme et al. (2011) found, in a greenhouse experiment, that females of the grass-specialist RWW laid double the amount of eggs in AMF-inoculated rice plants, an effect they speculated was caused by AMF-mediated increases in plant nutrient concentrations. In light of these prior results with RWW, we explored the hypothesis that colonization of roots by AMF would reduce the resistance of rice to the RWW in the field and greenhouse experiments. Then, in light of new results, we addressed a second hypothesis that AMF colonization might reduce the resistance of rice to other pest organisms such as FAW and ShB under greenhouse conditions. We asked the following questions: (1) Does AMF inoculation reduce rice resistance against a root- and foliar-feeding herbivore in the field and greenhouse? (2) Does AMF inoculation affect resistance to a fungal pathogen? (3) Does AMF inoculation increase plant biomass? (4) Does AMF inoculation influence the nutritional status of rice plants? To answer these questions, we carried out a series of field and greenhouse experiments in rice by manipulating the availability of AMF (inoculated and non-inoculated plants) using a commercial inoculum containing six AMF species from the Glomeraceae family. We found that the performance of insects and the pathogen on rice was enhanced when plants were colonized by AMF, which was consistent with results from Cosme et al. (2011); however, this susceptibility was not correlated with changes in plant nutritional status.

## Materials and Methods

### Study System: Plants, Fungi, and Insects

To study plant-AMF-herbivore and plant-AMF-pathogen interactions, we used two commercial varieties of rice as the host plant. ‘Lemont’ and ‘Cocodrie’ are high-yielding, early-maturing, conventional varieties developed at the Texas A&M University Agricultural Research and Extension Center (Beaumont, TX, United States) and the Louisiana State University Agricultural Center (LSU AgCenter) H. Rouse Caffey Rice Research Station (Crowley, Acadia, LA, United States), respectively (Bollich et al., 1985; Linscombe et al., 2000). ‘Cocodrie’ is a susceptible variety grown widely in the southern U.S. ‘Lemont’ is not widely grown currently but was chosen because it had been used in previous studies of rice-AMF interactions (Dhillion, 1992). Seeds of rice were kindly provided by the breeding and foundation seed program at the LSU AgCenter H. Rouse Caffey Rice Research Station. ‘Lemont’ was used for experiments in 2012 and ‘Cocodrie’ for experiments in 2013.

A commercial inoculum prepared *in vivo* to contain only AMF propagules (ECOVAM^TM^ VAM Endo Granular, Horticultural Alliance Inc., Sarasota, FL, United States) was used to promote and establish symbiosis with the host plants in the field and greenhouse experiments. The inoculum contained six species of AMF (*Rhizophagus irregularis*, *Funneliformis mosseae*, *Glomus deserticola*, *Rhizophagus fasciculatum*, *Sclerocystis dussii*, and *Glomus microaggregatum*) and consisted of spores, hyphae and colonized root fragments. All AMF species were originally obtained from the International Culture Collection of (Vesicular) AMF (INVAM, West Virginia University, United States). The AMF propagules were carried in an inert-like material consisting of a uniform mixture of zeolite, pumice, vermiculite, perlite, and attapulgite. According to the supplier, quantification of the number of spores per gram of inert material was accomplished by the wet sieving and decanting method of Gerdemann and Nicolson (1963) followed by sucrose gradient centrifugation according to the modification proposed by Schenck (1982). For the extraction of spores, 20 g of inert material was blended for 10 s in one liter of tap water. Counting was carried out under an optical microscope using a counting slide of 1 mL. The formulated material contained an average of 132 spores of AMF (all species) per gram, in addition to hyphae and colonized root fragments.

The RWW is the most destructive insect pest of rice in the United States (Stout et al., 2002; Tindall and Stout, 2003; Hamm et al., 2010). RWW adults feed on young rice leaves, producing longitudinal scars. However, this form of injury is not economically important; rather, the larvae have a strong impact on plant yields when they feed on roots of flooded rice (Cosme et al., 2011). Adult rice water weevils were collected from rice fields at the H. Rouse Caffey Rice Research Station 24 h prior to conducting greenhouse experiments. Field experiments relied on natural infestations of RWWs, which are abundant at the field site. Weevils were maintained in glass jars with freshly cut rice leaves and water until use. Before starting the experiment, weevils were captured in copula or sexed under a dissecting microscope in order to ensure equal numbers of males and females.

The FAW is a sporadic pest of rice that causes harm by consuming aboveground portions of rice with its chewing mouthparts. Adult female armyworms oviposit a large number of eggs on leaves, which give rise to larvae that begin to feed on leaves (Stout et al., 2009). Larvae of the FAW used in these experiments were obtained from a colony maintained continuously on meridic diet in a laboratory. The colony originated from larvae collected in rice fields near Crowley, LA, in 2011. Genetic variability and vigor of the colony were maintained annually with field-collected larvae. The diet used for rearing of larvae was a commercial formulation designed specifically for this species (Southland Products Incorporated, Lake Village, AR, United States). Pupae were placed in buckets containing vermiculite, wax paper as a substrate for oviposition, and two dental rolls soaked in a mixture of honey and beer (150 ml honey-150 ml beer- 300 ml water-12 g ascorbic acid) and covered with cheesecloth. After emergence, adults mated and females oviposited eggs onto the cheesecloth, which were collected daily and placed in 8-cell trays (Bio-Serv, Frenchtown, NJ, United States) with a moistened cotton ball and sealed with lids. When neonates began to emerge, they were placed in cups supplied with artificial diet. Larvae were maintained on meridic diet until use for feeding assays. The colony was maintained under controlled environmental conditions (L14: D10, 28 ± 2°C, 38 ± 2% R.H).

*Rhizoctonia solani* (Basidiomycete), the causal agent of ShB of rice, is a soilborne pathogen with a wide host range. The disease caused by this organism in rice usually develops after the tillering stage of rice growth, and initial infection appears on the stem near the water line as oval lesions, which dry and turn tan (Lee and Rush, 1983). The fungal isolate LR172 of the ShB pathogen used in this study was originally isolated in 1972 from a naturally infected rice plant (cv. ‘Lebonnet’) in Louisiana. LR172 was generously provided by D. Groth (LSU AgCenter H. Rouse Caffey Rice Research Station) and maintained on potato dextrose agar (PDA). Mycelial growth and sclerotia production were typical of *R. solani*. The isolate of *R. solani* was examined for mycelial growth with a compound microscope (Olympus CH2, Pittsburgh, PA, United States). A verified isolate of *R. solani* was subcultured by placing sclerotia in the center of a 9-cm-diameter petri dish filled with PDA medium to produce active mycelia and grown at room temperature (22–25^°^C) under continuous light. These cultures were used to prepare agar blocks of 5-day-old cultures inoculation.

### Experimental Design

#### Evaluating Effects of AMF on RWW Performance (Field Study)

To evaluate whether inoculation of rice plants with AMF affects the resistance of rice plants to *L. oryzophilus*, three small-plot field experiments were conducted during the 2012 and 2013 growing seasons at the LSU AgCenter H. Rouse Caffey Rice Research Station (Crowley, Acadia Parish, LA, United States). In 2012, one experiment, referred to as Experiment-1 (Exp-1) was conducted; in 2013, two experiments, Experiment-2 (Exp-2) and Experiment-3 (Exp-3) (**Table 1**), were conducted. Each experiment comprised three treatments. For the first treatment (F, fungicide) rice seeds were treated with a mixture of the fungicides Maxim 4FS (fludioxonil, 4.16 mg a.i. 300 g^-1^ of seeds; Syngenta Crop Protection, Greensboro, NC, United States), Apron XL 3LS (mefenoxam, 26.33 mg a.i. 300 g^-1^ of seeds; Syngenta Crop Protection, Greensboro, NC, United States) and Dynasty (azoxystrobin, 20.79 mg a.i. 300 g^-1^ of seeds; Syngenta Crop Protection, Greensboro, NC, United States) and planted in soil with sterilized AMF inoculum. Rice seeds were treated with a mixture of fungicides before planting to eliminate the presence of any fungi from experimental plots. For the second treatment (NM, nonmycorrhizal), rice seeds were sown in soil with sterilized AMF inoculum. The sterilized inoculum was used in nonmycorrhizal plots to control for the possibility that inert ingredients in the commercial inoculum altered soil properties. For the F and NM treatments, commercial inoculum was sterilized by autoclaving for 60 min at 120^°^C to destroy living AMF inoculum. For the third treatment (M, mycorrhizal), rice seeds were planted in soil inoculated with live AMF. For all three experimental treatments, rice plants were grown from seeds in the field; thus the soil was not sterilized and likely contained native AMF. Sterilized mock or live AMF inoculum was applied on the surface of the soil and gently raked in to incorporate the live or mock inoculum into the upper 2.5 cm of the soil. Experiments were laid out in a randomized complete block design (RCBD; in Exp-1) or in a completely randomized design (CRD; in Exp-2 and 3) with a total of eight and ten blocks (replications) per treatment per experiment for 2012 and 2013, respectively.

**Table 1:** Planting and sampling dates for three field experiments conducted in 2012 and 2013 for evaluating the effects of arbuscular mycorrhizal fungi on the performance of rice water weevil in rice plants.

Year	Trial	Planting date	Flooding date	Larval sampling dates (cores)
2012	Experiment-1	17^th^ April	30^th^ May	15^th^ June and 20^th^ June
2013	Experiment-2	4^th^ April	30^th^ May	19^th^, 24^th^ June and 2^nd^ July
	Experiment-3	6^th^ June	24^th^ June	15^th^, 22^th^ and 29^th^ July

Rice was hand-seeded on the dates specified in **Table 1** at a rate of 10 g of seeds per plot. Plots measured 0.762 m × 0.762 m. A soil sample was collected from the plots before seeding in 2013 and sent for analysis to the LSU AgCenter Soil Testing & Plant Analysis Laboratory (STPAL, LSU, Baton Rouge, LA, United States). The principal chemical properties of the soil are reported in Supplementary Table S1. Each plot was inoculated with 1.5 kg (2012) or 2 kg (2013) of sterilized AMF inoculum (F and NM) or live inoculum (M). The inoculum amounts used in 2012 and 2013 corresponded to approximately 200 and 260 thousand AMF spores per plot, respectively. To avoid the spread of AMF inoculum from plot to plot during irrigation, plots were surrounded by an enclosure constructed of metal roofing flashing 20 cm high and held in place by pushing into the soil before planting. Plots were flushed with well water as necessary for the first month after seeding to establish stands of rice. We did not incorporate small filtrate aliquots of AMF inoculum into plots because we assumed that the large volumes of flooding water were sufficient to allow some homogenization among treatments in terms of water-soluble microflora, whereas the loose AMF spores, which are denser than water, were expected to remain precipitated. After allowing the plants to grow for approximately 1 month, a permanent flood was applied on the dates specified in **Table 1**. Plants possessed 4-5 leaves (early tillering) at permanent flooding. Metal flashing was removed after flooding. Plots in these experiments were not fertilized.

After natural infestation, densities of RWW larvae and pupae were determined by taking root/soil core samples from each plot (Stout et al., 2001). The core sampler was a metal cylinder with a diameter of 9.2 cm and a depth of 7.6 cm attached to a metal handle (Supplementary Figure S1). Core sampling was conducted twice for all experiments between 3 and 5 weeks after permanent flood. Dates of core samplings are shown in **Table 1**. For each sampling date, two (2012) or three (2013) core samples were taken from each plot. Core samples were placed into a 40-mesh screen sieve bucket to wash the soil and larvae from roots, buckets were placed into basins of salt water, and larvae and pupae were counted as they floated to the water surface (N’Guessan et al., 1994). RWW counts from two to three core samples per plot per sampling date were averaged to obtain an average number of larvae/pupae per core sample.

In order to confirm if the inoculum enhanced the abundance of AMF living in rice roots in Exp-2 and 3, the percentage of the root system containing AMF colonization was determined by observation of sub-sampled root fragments as described below. For Exp-2, the percentage of root fragments colonized by AMF was evaluated two times during plant development, before and after flood. For Exp-3, this parameter was evaluated one time after the flood was established. On May 15th (41 dai) and June 7th (64 dai), 12 root samples from Exp-2 were randomly collected and analyzed from four plots of each treatment group per sampling date. The same number of root samples from Exp-3 were collected and analyzed from four plots of each treatment group on July 8th (32 dai). Sampling in Exp-2 and 3 was conducted by taking 9.2 cm diameter soil-root cores adjacent to plants. Each soil-root core (2–4 plants) was placed in plastic bags (one core per bag) and taken to the laboratory to be processed as described below for root staining. For the purpose of this study, one core represented one plant sample. A list of the experiments conducted in 2012 and 2013 are summarized in Supplementary Table S2.

#### Evaluating Effects of AMF on Plant Resistance to RWW (Greenhouse Study)

To further evaluate whether AMF inoculation alters the resistance of rice to *L. oryzophilus*, two choice experiments (RWW1 and RWW2) were conducted in the summer of 2013 in a greenhouse on the campus of Louisiana State University, Baton Rouge, LA, United States. For each experiment, two treatments were employed, namely mycorrhizal (M) and nonmycorrhizal plants (NM; control). All plants were grown in 2 liter round (15 cm diameter) plastic pots (Hummert International, Earth City, MO, United States) filled with a sterilized soil mix (2:1:1, soil: peat moss: sand), to which 50 g of AMF inoculum (corresponding to approximately 6500 AMF spores) or 50 g sterilized inoculum were added. For all greenhouse experiments, the soil substrate was sterilized by autoclaving for 60 min at 120^°^C to eradicate the indigenous AMF. The AMF inoculum was mixed with the soil, and rice seeds were sown directly into pots. Plants were maintained under greenhouse conditions with temperatures ranging from 25 to 35^°^C and ambient lighting. Plants were maintained in large wooden basins lined with heavy black plastic pond liner to hold flood waters when necessary as indicated in Stout and Riggio (2002). As for the field study, we assumed that flooding waters were suffice to allow some homogenization of water-soluble microflora. Approximately 10 days after planting, seedlings were thinned to a density of two or three plants per pot (RWW1 and RWW2, respectively). Experiments were conducted using 2-week-old plants (3-leaf stage). Because these experiments were conducted with rice at an early stage of growth, additional fertilizer was not necessary for adequate plant growth.

To initiate the choice experiments, two pots of each treatment were placed into each of seven (RWW1) or six (RWW2) infestation cages (Supplementary Table S2 and Supplementary Figure S2). Cages were set in the greenhouse basins and basins were flooded to a depth of ∼20 cm. Infestation cages were cylindrical wire frames (46 cm diameter × 61 cm tall) covered with a mesh fabric screening. After flooding, weevils were released into cages at a density of three weevils per plant (24 and 36 weevils per cage in RWW1 and RWW2, respectively) and allowed to feed, mate, and oviposit on plants of both treatments for 5 days. After that, pots were removed from cages and weevils were discarded.

The resistance of M and NM plants to *L. oryzophilus* was evaluated by counting first instars as they emerged from eggs laid in leaf sheaths of plants. Procedures for estimating larval densities were adapted from Stout and Riggio (2002). Briefly, after the 5-day adult infestation, plants for each pot were removed from the soil, washed free of soil, and placed individually in water in clean test tubes. Test tubes were labeled, arranged in a test tube rack, and placed in a growth chamber (30^°^C, 14:10 L:D). Using this method, weevils that infest plants hatch from eggs, emerge from leaf sheaths and settle on the bottom of the test tubes (Heinrichs et al., 1985). Larvae were removed by shaking roots free of larvae and then pouring water from test tubes into a petri dish for counting. After that, plants were placed back into the test tubes, and tubes were refilled with fresh water. Larva counts were started 3 days after placing plants in the tubes, and larvae were counted daily until no additional larvae were found for two consecutive days.

The percentage of root fragments colonized by AMF was measured in RWW2. Root samples from 5 plants of each mycorrhizal treatment were sampled on July 18th, 31 dai. A total of 10 plant samples were collected from this experiment.

#### Evaluating Effects of AMF on Plant Resistance to FAW (Laboratory Study)

To assess whether AMF inoculation influences resistance of rice to *S. frugiperda*, three laboratory feeding assays were conducted in 2012 (FAW1) and 2013 (FAW2 and FAW3). To this end, we cut leaf material from greenhouse-grown plants with or without AMF inoculum to determine *S. frugiperda* larval growth. ‘Lemont’ and ‘Cocodrie’ rice plants were grown under two treatments, namely M and NM. Plants were grown in the greenhouse as previously described. Six rice seeds were planted in each pot and thinned to three plants immediately before starting feeding assays for FAW1, FAW2, and FAW3 (Supplementary Table S2). Plants from which leaf material was taken were 3 weeks old and possessed three or four leaves. Because these experiments were conducted with rice at an early stage of growth, additional fertilizer was not necessary for adequate plant growth.

To initiate the assays, larvae of 4–5 days in age were selected from meridic diet and stage-synchronized at head capsule slippage. Synchronized larvae were starved for 3 h to ensure that their guts were voided before their masses were determined using an analytical balance (model XS105, Mettler-Toledo LLC, Columbus, OH, United States). Larvae with similar masses were used in these experiments. Feeding assays were conducted in 9 cm plastic petri dishes lined with moistened cotton batting to maintain turgor in excised tissues (Supplementary Figure S3). Youngest fully-expanded leaves were removed from plants of each treatment group using scissors, transported on ice to the laboratory, cut into ca. 2 cm pieces and placed in petri dishes. Weighed larvae were placed together in petri dishes with foliage and allowed to feed on excised leaf material for 4 days (FAW1), 7 days (FAW2), or 10 days (FAW3). Larvae were observed daily to ensure they were not food-limited and leaves were changed every other day, but in later larval stage the leaves were changed daily. After ending the feeding assay, larvae were starved for 3 h to ensure that the larval gut was emptied before final mass was determined and recorded. For each experiment, 15 larvae (replicates) were used for each treatment for a total of 28, 30, and 30 observations for FAW1, FAW2, and FAW3, respectively (insects that died during feeding assays were excluded).

The percentage of root fragments colonized by AMF was measured in FAW2. To this end, root samples from 5 plants of each treatment were sampled on May 24th, 35 dai in 2013, and processed as described below. For the experiment FAW3 described here, RWW1 described above, and ShB1 described below, only one assessment of AMF colonization was conducted as these three experiments were planted at the same time and the inoculation success had been previously confirmed. From a total of 100 pots planted (50 M and 50 NM) in these three experiments, five M and five NM plants were sampled on Jun 27th, 36 dai in 2013. A total of 20 plant samples were collected from the four experiments.

#### Evaluating Effects of AMF on Plant Resistance to Rice Sheath Blight (Greenhouse Study)

To investigate whether AMF inoculation influences susceptibility of rice to infection by the fungus *R. solani*, two experiments (ShB1 and ShB2) were conducted in the summer of 2013. To obtain uniform disease development, rice plants at late tillering growth stage (approximately 8-weeks-old) were used for inoculation with *R. solani*. As in previous experiments, M and NM treatment plants were set up in the greenhouse filled with sterilized soil mix. Six rice seeds were planted in each pot and thinned to five and three plants immediately before pathogen inoculation for ShB1 and ShB2, respectively (Supplementary Table S2). Plants in each pot were collectively considered an experimental unit (replication). Fifteen pots of each treatment group were used for each experiment and arranged in a completely randomized design in greenhouse basins. Because these experiments were conducted with rice at late stage of growth, additional fertilizer was necessary for adequate plant growth. Urea (46% N) was applied at 0.5 g (134 kg N/ha) per pot in all pots (ShB1 and ShB2). Fertilizer was applied twice at 20 days and 40 days after planting.

Agar blocks (0.5 cm squares) of a 5-day-old culture of LR172 were cut from the outer growing area of culture plate using a pipette tip. Using forceps, one tiller of each plant, i.e., five or three tillers in each pot, was inoculated with *R. solani* by placing the mycelial agar block beneath the leaf sheath, ensuring that mycelia were in contact with the plant. The leaf sheath and agar block were covered immediately with aluminum foil as described by Park et al. (2008). Inoculated plants were maintained in the greenhouse, where relative humidity was favorable for the growth of ShB. When typical lesions started to appear 3 days after inoculation (dai), the aluminum foil was removed to allow for disease development (Supplementary Figure S4). Susceptibility of rice plants to ShB was evaluated 7 dai for each tiller by counting the number of lesions and measuring the lesion length of each inoculated plant. For each plant, measurements of lesion length were used to derive the maximum lesion length and the mean lesion length.

#### Processing and Quantification of Mycorrhizal Colonization

The trypan blue method of Koske and Gemma (1989) for root staining was used for quantification of mycorrhizal colonization with some modifications. Clearing and staining procedures require root samples to be washed from soil to remove all soil particles and then separating root and shoot tissues. For subsampling, roots of each plant were cut into 2-cm-long segments and placed in tissue processing cassettes (Ted Pella, Redding, CA, United States). At least 200 small root pieces per root sample were cleared in 10% KOH at 90^°^C for 20 min in a water bath. Clear pieces of roots were rinsed 5X with tap water to remove KOH, and roots were immersed in 2% HCl at room temperature for 10–15 min to ensure the roots were adequately acidified for staining. Cassettes containing roots were immediately stained with 0.05% trypan blue (Sigma-Aldrich, St. Louis, MO, United States) by incubation overnight and then transferred to vials containing lactoglycerol at 4^°^C to allow excess stain to leach out of the roots. Stained root samples were stored in destaining lactoglycerol solution for 48 h before being mounted in the same solution on a microscopic slide.

In order to quantify the abundance of AMF living in rice roots, the 2-cm-long root fragments were mounted after staining on microscopic slides as previously described (McGonigle et al. (1990). Five microscope slides, each containing ten stained randomly selected root fragments, were prepared from each plant sample. The random selection of root fragments is representative for the whole root system as it was often not possible to disentangle the root types. A total of 50 stained root segments per sample were examined with a compound microscope (Olympus CH2, Tokyo, Japan) at 40× magnification in order to confirm the levels of AMF colonization. Root fragments that contained blue-stained AMF structures such as intraradical aseptate hyphae linked to either fungal arbuscules or vesicles/spores were scored as colonized by AMF (Supplementary Figure S5) (DeMars and Boerner, 1996). Percent of root fragments with AMF colonization was averaged per treatment for the analyzed experiments. Photos of AMF structures on mycorrhizal colonized roots were taken using a microscope-mounted 5.0-megapixel digital camera (Leica DFC480, Cambridge, United Kingdom).

#### Evaluating Effects of AMF on Plant Biomass

To determine the effect of AMF on plant biomass, rice samples were collected from Exp-2 and from a separate greenhouse experiment (PB1) conducted in 2013 using previously sterilized field soil from the LSU AgCenter H. Rouse Caffey Rice Research Station. For PB1, NM and M treatments were established with 12 replications for each treatment as described previously (Supplementary Table S2). Entire plants were collected on June 18th from Exp-2 and on September 24th for PB1 at 75 and 30 dai, respectively. Pots for PB1 were not fertilized. Soil was washed from roots, and the shoots and roots were separated and blotted dry with a paper towel. Fresh weights of shoots and roots were recorded, and plant material was dried in an oven (60^°^C for 1 week) and reweighed (shoot and root dry weight) to calculate plant dry biomass as well as the ratio of root dry weight (RDW)/shoot dry weight (SDW).

#### Evaluating Effects of AMF on Plant Nutritional Status

To evaluate whether AMF inoculation affected the concentrations of nutrients in leaves and roots of rice, above- and belowground plant tissue samples from each of the treatments in Exp-1, Exp-2 and PB1 were collected on May 30th, June 18th, and September 24th at 43, 75 and 28 dai, respectively. Plant material was washed and transported to the laboratory. Samples were dried in an oven at 60^°^C for 1 week, ground in a Wiley mill (Thomas Wiley^®^ Mini-Mill, Mexico) and submitted to the LSU AgCenter’s Soil Testing & Plant Analysis Laboratory (STPAL, LSU, Baton Rouge, LA, United States) to determine nutrient concentrations in shoot and root tissues. The STPAL determined N and C concentrations by dry combustion using a LECO TruSpec^TM^ CN analyzer (LECO Corp., St. Joseph, MI, United States), while the concentrations of the remaining nutrients (Ca, Mg, S, P, K, Al, B, Cu, Fe, Mn, Na, and Zn) were determined by inductively coupled plasma (ICP) analysis.

#### Statistical Analyses

Data were analyzed using SAS 9.4 (SAS Institute, 2014). The effects of AMF inoculation on rice plant responses for each experiment were analyzed separately by one-way analysis of variance (ANOVA) using PROC MIXED. For the RWW field experiments, effects of AMF inoculation on average number of larvae/pupae per core sample were analyzed as appropriate for a RCBD with treatment (F, NM, or M) as a fixed effect and block (replication) as a random effect for Exp-1 or CRD with treatment (F, NM, or M) as fixed effect for Exp-2 and Exp-3. For the RWW choice experiments, data were analyzed with treatment as a fixed effect and infestation cages (replication) as a random effect. For the FAW experiments, weight gain (final weight – initial weight) was the response variable, treatment was a fixed effect, and experiment was a random effect. For ShB experiments, disease ratings (lesion length and numbers of lesions) from five and three individual plants in each pot, respectively, were averaged as a single replication. The two experiments were analyzed independently with lesion length and number of lesions as dependent variables with treatment considered as a fixed effect. The data on AMF colonization were analyzed based on the percentage of root fragments colonized (see above) for Exp-2, Exp-3, RWW2, FAW2, and FAW3/RWW1/ShB1 experiments. Data for SDW and RDW were analyzed with the two treatments (M and NM) as fixed effects. For nutritional analyses, data for each nutrient (N, P, K, and C) were analyzed separately. Means were separated using the least significant difference (LSD) test in each of the experiments when there was a significant difference between treatments.

## Results

### Root Colonization by AMF

The microscopic analyses of root fragments collected from M, NM or F treated rice plant samples in experiments Exp-2, Exp-3, RWW2, FAW2 and in a random sampling of FAW3, RWW1 and ShB1 combined (see section “Materials and Methods” above) confirmed that AMF inoculation significantly enhanced the percentage of root fragments colonized by AMF in relation to the non-inoculated controls. This was observed in greenhouse grown plants and in field grown plants (**Table 2** and Supplementary Figure S5); except in Exp-2 prior flooding at 41 dai, in which the enhanced percentage of root fragments colonized by AMF was only apparent in M plants compared with the non-inoculated plants. For both field experiments (Exp-2 and Exp-3), we detected a small percentage of fragments colonized by AMF in the non-inoculated plants or in the plants treated with fungicide (**Table 2**), probably due to native AMF already present in soil. Overall, although the percentages of root fragments colonized by AMF in rice were generally low, our data confirm that inoculation with AMF enriched the abundance of AMF living in rice roots grown under greenhouse and field conditions.

**Table 2:** Percentage (%) of root fragments colonized by arbuscular mycorrhizal fungi (AMF) in rice plants.

Treatments	Root fragments colonized by AMF (%)		
Field 2013 (Mean of 4 samples each)	Exp-2 (41 dai^1^) Mean ± SE	Exp-2 (64 dai) Mean ± SE	Exp-3 (32 dai) Mean ± SE
Fungicide (F)	1.5 ± 0.95b	0.5 ± 0.50b	0.5 ± 0.50b
Nonmycorrhizal (NM)	4 ± 1.83ab	1.5 ± 0.95b	3 ± 1.29b
Mycorrhizal (M)	9 ± 2.08b	6 ± 2.16a	7 ± 1.29a
***F*_2,9_**	5.10	4.41	9.00
***P-value***	0.033	0.046	0.007

**Greenhouse 2013 (Mean of 5 samples each)**	**RWW2 (31 dai) Mean ± SE**	**FAW2 Mean ± SE**	**FAW3/RWW1/ ShB1 (36 dai) Mean ± SE**

Nonmycorrhizal (NM)	0.8 ± 0.49b	0.4 ± 0.40b	0 ± 0b
Mycorrhizal (M)	8.4 ± 2.48a	11.6 ± 1.72a	13.6 ± 1.72a
***F*_1,8_**	9.03	40.20	62.49
***P-value***	0.017	0.0002	< 0.0001

*The percentage of colonized root fragments was determined from two field experiments (Experiment-2, Experiment-3), and from five greenhouse experiments (FAW2, RWW2, and from the combined experiments FAW3/RWW1/ShB1). Means ± standard errors are shown (*n* = 4 or 5 for field and greenhouse, respectively). Different letters indicate significant differences between mycorrhizal levels within each mycorrhizal treatments according to Least Significant Difference mean comparisons (*P* < 0.05; LSD). The F, NM, and M refer to AMF treatments of F: rice seeds + fungicides + sterilized AMF, NM: rice seeds + sterilized AMF, and M: rice seeds + live AMF. **^*1*^**dai, days after inoculation*.

### Effects of AMF Inoculation on RWW Performance in the Field

**FIGURE 1:**
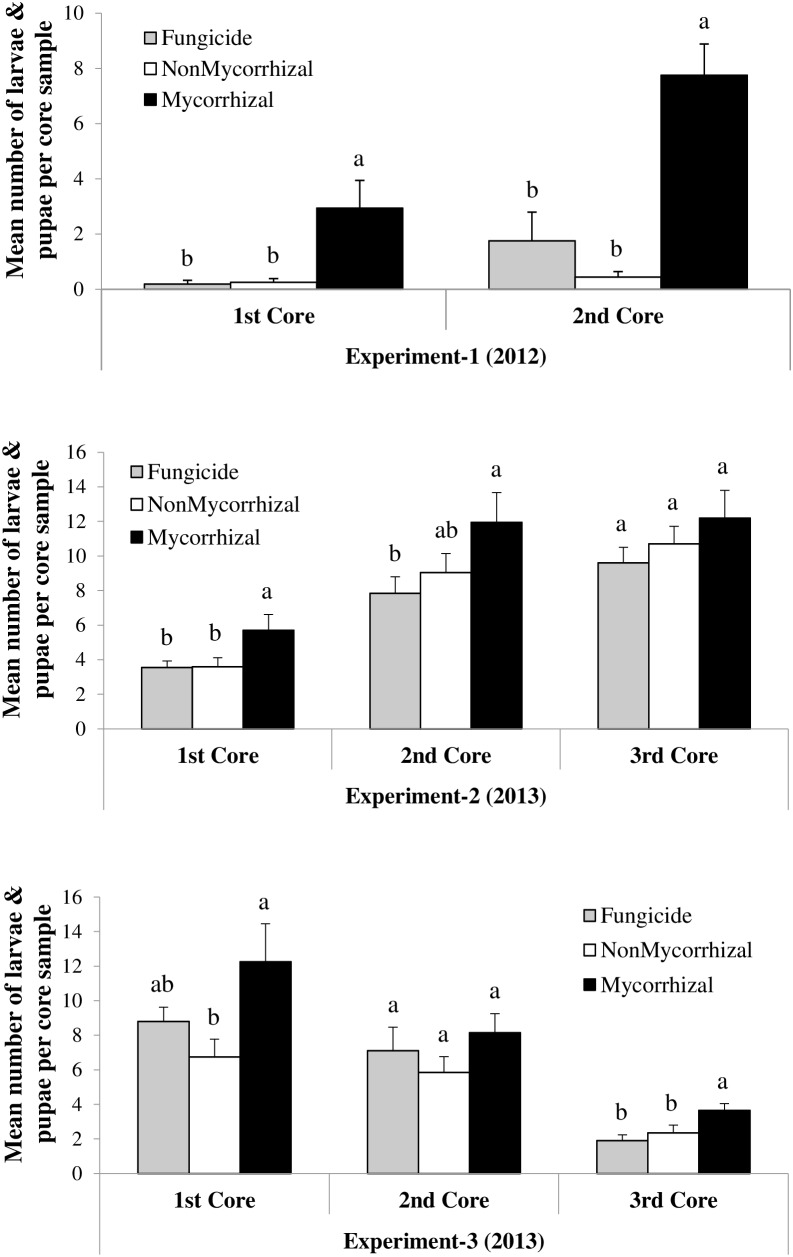
Effects of arbuscular mycorrhizal fungi treatments on the densities (larvae and pupae per core sample) of *Lissorhoptrus oryzophilus* (± SE) in three field experiments (Experiment-1, Experiment-2, and Experiment-3) during 2012 and 2013. Fungicide: rice seeds + fungicides + sterilized AMF, NonMycorrhizal: rice seeds + sterilized AMF, Mycorrhizal: rice seeds + live AMF. Bars and lower case letters at the column head indicate that means differ significantly (LSD, *P* ≤ 0.05).

Under field conditions, the susceptibility of AMF-inoculated rice plants to RWW was measured by the densities of RWW larvae and pupae compared with that of rice plants treated with sterilized inoculum or with fungicides and sterilized inoculum (**Figure 1**). For Exp-1, we observed a significant positive impact of AMF inoculation on rice susceptibility to RWW larvae and pupae on both core sampling dates (June 15: *F*_2,14_ = 7.45, *P* = 0.0063; June 20: *F*_2,14_ = 21.06, *P* < 0.0001) (**Figure 1**). The highest immature densities were found in plots of plants inoculated with AMF on both sampling dates, whereas densities were lowest, at nearly equal numbers, in plots inoculated with sterilized inoculum or with fungicide and sterilized inoculum. Also, densities increased over time: weevil densities were lowest at 15 (core 1) days after permanent flood and highest at 20 (core 2) days after permanent flood. Increases in RWW densities in plots of AMF-inoculated plants ranged from 91.4% in core 1 (2.94 ± 1.01 to 0.25 ± 0.13, mean ± SE) to 94.3% in core 2 (7.75 ± 1.13 to 0.44 ± 0.19, mean ± SE) when compared to NM plants. For Exp-2, the AMF-mediated susceptibility of rice to RWW larvae and pupae was only significant in the first core sampling, while in the second and third core samplings the enhanced susceptibility was not apparent (June 19: *F*_2,18_ = 4.15, *P* = 0.0331; June 24: *F*_2,18_ = 2.64, *P* < 0.0990; July 2: *F*_2,18_ = 1.26, *P* = 0.3074). As in Exp-1, weevil densities in Exp-2 increased with sampling date, being lowest at 19 (core 1) days after permanent flood, intermediate at 24 (core 2) days, and highest at 32 (core 3) days after permanent flood (**Figure 1**). The increase in weevil densities in plots of AMF-inoculated plants in core 1 was 37% (5.70 ± 0.92–3.60 ± 0.52, mean ± SE) when compared to NM control plants. In second and third core samplings, increases were not meaningful with 24.2% (11.95 ± 1.72 to 9.05 ± 1.09, mean ± SE) and 12.3% (12.20 ± 1.60 to 10.70 ± 1.02, mean ± SE), respectively. In Exp-3, densities of RWW were significantly higher in AMF-inoculated plants in the first and third core samplings (July 15: *F*_2,18_ = 4.32, *P* = 0.0293; July 29: *F*_2,18_ = 6.20, *P* = 0.0090) but not in the second core sampling (July 22: *F*_2,18_ = 1.11, *P* < 0.3497), compared with both non-inoculated control treatments. Unlike previous experiments, weevil densities in Exp-3 decreased with sampling date: weevil densities were highest at 21 (core 1), intermediate at 28 days (core 2), and lowest at 35 (core 3) days after permanent flood. Increases in RWW densities in plots of AMF-inoculated plants ranged from 45% in core 1 (12.25 ± 2.20 to 6.75 ± 1.02, mean ± SE) to 36% in core 3 (3.65 ± 0.39 to 2.35 ± 0.45, mean ± SE) when compared to NM control plants. Overall, the inoculation of rice plants with AMF enhanced the susceptibility of rice to RWW in all three field experiments (Experiment-1: *F*_2,14_ = 26.44, *P* < 0.0001; Experiment-2: *F*_2,18_ = 5.59, *P* = 0.013; Experiment-3: *F*_2,18_ = 7.00, *P* = 0.0056).

### Effects of AMF Inoculation on Plant Resistance to RWW in the Greenhouse

**FIGURE 2:**
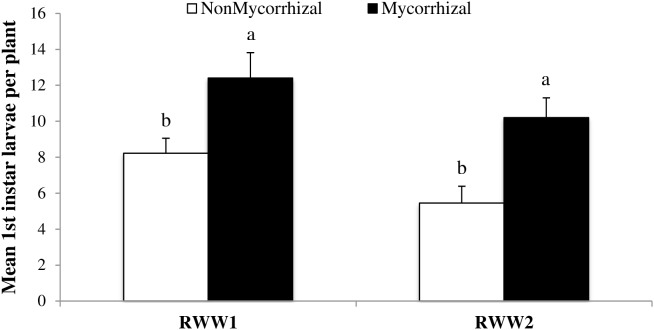
Mean number of *Lissorhoptrus oryzophilus* larvae per plant (± SE) in a greenhouse experiment using mycorrhizal (M) and nonmycorrhizal (NM) rice plants of the variety ‘Cocodrie.’ Plants were infested with pairs of rice water weevil adults to feed on each plant for 5 days. NonMycorrhizal: rice seeds + sterilized AMF, Mycorrhizal: rice seeds + live AMF. Bars and lower case letters at the column head indicate that means differ significantly (LSD, *P* ≤ 0.05).

Arbuscular mycorrhizal fungi colonization can increase rice susceptibility to oviposition by RWW females (Cosme et al., 2011), but it was yet unclear whether this affects subsequent developmental stages. In order to address this question, we assessed the number of RWW first instars emerging from rice plants subjected to oviposition under controlled conditions. In two independent experiments (RWW1 and RWW2) inoculation with AMF of rice roots significantly increased the numbers of RWW first instars emerging from M treated rice plants (**Figure 2**; RWW1: *F*_1,48_ = 6.99, *P* = 0.0110; RWW2: *F*_1,65_ = 13.66, *P* = 0.0005). Numbers of RWW first instars emerging from M rice plants were 34 and 47% greater in RWW1 (12.39 ± 1.43 to 8.21 ± 0.95, mean ± SE) and in RWW2 (10.19 ± 1.11 to 5.44 ± 0.95, mean ± SE), respectively, compared to NM control plants. Therefore, AMF inoculation also has a positive impact on the performance of early stages of RWW.

### Effects of AMF Inoculation on FAW Growth

**FIGURE 3:**
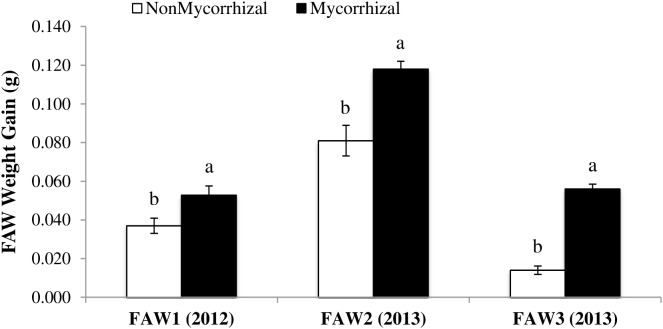
Weight gain (g ± SE) of *Spodoptera frugiperda* larvae fed on rice leaves from nonmycorrhizal (NM) and mycorrhizal (M) plants in lab studies during 2012 and 2013. Feeding assays were performed for 4, 7, and 10 days with larvae of 4 to 5 days old. NonMycorrhizal: rice seeds + sterilized AMF, Mycorrhizal: rice seeds + live AMF. Bars and lower case letters at the column head indicate that means differ significantly (LSD, *P* ≤ 0.05).

To understand whether the increase in susceptibility of rice plants colonized by AMF is specific to RWW, we assessed the impact of inoculation with AMF on growth of FAW larvae. For all three FAW experiments, FAW larvae gained more weight when fed leaf material from plants inoculated with AMF compared with larvae fed leaf material from NM plants (FAW1: *F*_1,26_ = 6.72, *P* = 0.015; FAW2: *F*_1,28_ = 16.82, *P* = 0.0003; FAW3: *F*_1,28_ = 159.24, *P* < 0.0001) (**Figure 3**). Increases in larval growth on M rice plants ranged from 30.2% in FAW1 (0.053 ± 0.004 to 0.037 ± 0.003, mean ± SE), 31.4% in FAW2 (0.118 ± 0.004 to 0.014 ± 0.007, mean ± SE) to 75% in FAW3 (0.056 ± 0.003 to 0.014 ± 0.002, mean ± SE) compared with the NM control plants. These results show that the impact of AMF on rice susceptibility to herbivores affects aboveground herbivores as well as root feeding herbivores.

### Effects of AMF Inoculation on Plant Resistance to Sheath Blight

**FIGURE 4:**
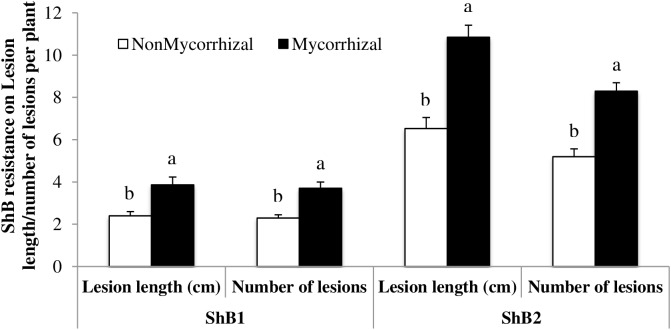
Rice sheath blight disease variables (lesion length and number of lesions) measured after inoculation with isolate LR172 of *Rhizoctonia solani* in mycorrhizal and nonmycorrhizal rice plants in greenhouse experiments in the summer 2013. NonMycorrhizal: rice seeds + sterilized AMF, Mycorrhizal: rice seeds + live AMF. Bars and lower case letters at the column head indicate that means differ significantly (LSD, *P* ≤ 0.05).

In order to determine whether AMF-induced rice susceptibility also extends to pathogenic microorganisms, we analyzed the infection levels by ShB in rice stems. In two independent experiments, inoculation of rice roots with AMF significantly increased both measures of damage caused by ShB, i.e., lesion length (ShB1: *F*_1,28_ = 11.83, *P* = 0.0018; ShB2: *F*_1,28_ = 31.80, *P* < 0.0001) and numbers of lesions (ShB1: *F*_1,28_ = 17.06, *P* = 0.0003; ShB2: *F*_1,28_ = 34.27, *P* < 0.0001). Lesion length in M rice plants was 38% and 40% greater in ShB1 (3.86 ± 0.38 cm to 2.40 ± 0.20 cm, mean ± SE, *n* = 15) and ShB2 (10.85 ± 0.56 to 6.53 ± 0.52 cm, mean ± SE, *n* = 15), respectively, compared with lesion length in NM control plants. Similarly, the numbers of lesions in the two experiments were greater on M rice plants as compared to the NM plants (37% greater in ShB1: 3.67 ± 0.30 to 2.31 ± 0.14, mean ± SE, *n* = 15 and 38% greater in ShB2: 8.29 ± 0.39 to 5.16 ± 0.36, mean ± SE, *n* = 15). Leaves from M plants developed clear symptoms of infection at 3 days post-inoculation. At this time, only small necrotic spots were evident on NM plants. Lesions advanced aggressively on the leaves of mycorrhizal plants, and after 7 days post-inoculation these leaves were severely damaged (Supplementary Figure S4). Overall, these results show that AMF-induced rice susceptibility is also observed with an aboveground fungal pathogen (**Figure 4**).

### Effects of AMF Inoculation on Plant Biomass

In Exp-2, the shoot biomass of M rice plants differed significantly from the shoot biomass of rice plants treated with sterilized inoculum (NM) or with fungicides and sterilized inoculum (F) (*F*_2,6_ = 12.15, *P* = 0.008), ranging from 2.17 to 3.94 g (**Table 3**). The effect of AMF inoculation on root biomass and root-to-shoot ratio was not significant (**Table 3**). In 75-day-old rice plants, the SDW of M rice plants was 32.7% higher than the SDW of NM plants. In the PB1 experiment, M rice plants exhibited significantly higher shoot biomass than NM plants (*F*_1,11_ = 6.53, *P* = 0.027) (**Table 3**), ranging from 0.88 to 1.09 g (**Table 3**). As in Exp-2, neither root biomass nor root-to-shoot ratio of rice plants differed among the different AMF treatments (**Table 3**). The SDW of the 30-day-old rice plants was 19.3% higher in M plants as compared to NM plants (**Table 3**).

**Table 3:** Results from one-way ANOVA on the effect of arbuscular mycorrhizal fungi (AMF) on the shoot and root dry weight biomass and root: shoot ratio of 75 and 30 day-old rice plants from a field (Exp-2) and a greenhouse experiment (PB1) in 2013.

Treatments	Shoot DW (g)	Root DW (g)	Root DW/ Shoot DW
**Field 2013 (Exp-2)**	**Mean ± SE**	**Mean ± SE**	**Mean ± SE**

Fungicide (F)	2.17 ± 0.38b	1.02 ± 0.08b	0.50 ± 0.07a
Nonmycorrhizal (NM)	2.65 ± 0.48b	1.19 ± 0.27a	0.45 ± 0.04a
Mycorrhizal (M)	3.94 ± 0.36a	1.25 ± 0.21a	0.34 ± 0.08a
	(32.7%)^∗^	(4.8%)^∗^	(-32.4%)^∗^
***F*_2,6_**	12.15	0.38	2.15
***P-value***	**0.008**	0.699	0.198
**GH 2013 (PB1)**			
Nonmycorrhizal (NM)	0.88 ± 0.05b	0.51 ± 0.05a	0.57 ± 0.05a
Mycorrhizal (M)	1.09 ± 0.06a	0.60 ± 0.04a	0.56 ± 0.04a
	(19.3%)^∗^	(15.0%)^∗^	(-1.8%)^∗^
***F*_1,11_**	6.53	2.46	0.02
***P-value***	**0.027**	0.145	0.901

*DW = Dry Weight. Mean values followed by different letters within columns indicate a significant difference among treatments by Least Significant Difference mean comparisons (*P* < 0.05; LSD). Significant *P*-values are in bold. The F, NM, and M refer to AMF treatments of F: rice seeds + fungicides + sterilized AMF, NM: rice seeds + sterilized AMF, and M: rice seeds + live AMF. ^∗^The relative change (%) in root, shoot and ratio was calculated by dividing the difference of AMF and non-AMF by AMF treatment*.

### Effects of AMF Inoculation on Plant Nutritional Status

No effects of AMF inoculation on concentrations of plant nutrients were found in either the field experiment, Exp-2, which showed low levels of AMF colonization in the non-inoculated controls, or in the greenhouse experiment (PB1), which had a nonmycorrhizal control without AMF (Supplementary Table S3). Therefore, the increases in shoot biomass and susceptibility to pests in AMF-inoculated plants were not accompanied by increases in concentrations of N, P, K or C. (Supplementary Table S3).

## Discussion

Interactions among AMF and plants can alter the suitability of plants for herbivores and pathogens. These effects have been investigated in a number of systems (Gange and West, 1994; Pineda et al., 2010; Currie et al., 2011) but have not been extensively investigated in rice, one of the most important crops not only in the United States but also worldwide (Campos-Soriano et al., 2011; Cosme et al., 2011). In this study, we used a commercial formulation of AMF containing multiple species from the Glomeraceae family to investigate the effects of inoculation with AMF on rice resistance against two important herbivores and one important pathogen. These biotic interactions were investigated in a wetland rice system. It is widely recognized for wetland systems that, although AMF can live through the year and occur in all plant developmental stages, flooding strongly suppresses levels of AMF colonization of roots (Solaiman and Hirata, 1995, 1996, 1997; Miller and Bever, 1999; Miller and Sharitz, 2000; Purakayastha and Chhonkar, 2001). Previously observed colonization levels in wetland rice under flooded conditions have ranged from 4% at 14 dai (Cosme et al., 2011), 5% at 30 dai (Campos-Soriano et al., 2010), 2–12% at 60 dai (Solaiman and Hirata, 1995), 14–29% at 40 dai (Purakayastha and Chhonkar, 2001), and > 30% at 75 dai (Solaiman and Hirata, 1997). Such low levels of colonization by AMF in wetland rice have nonetheless been associated with significant impacts on plant growth and nutrition (Solaiman and Hirata, 1995, 1996, 1997; Purakayastha and Chhonkar, 2001). In addition to the suppressive effects of flooding on AMF colonization, not all tissues of rice roots are susceptible to AMF colonization. Previous studies have shown that only large lateral roots of rice are substantially susceptible to AMF colonization, whereas crown roots are generally poorly colonized and fine lateral roots are never colonized (Gutjahr et al., 2009, 2015a). Such specialization in colonization dilutes the levels of colonization in the whole root system. Thus, the low levels of colonization of rice roots by AMF observed using the sampling and staining techniques described in this study were not surprising. Despite the low levels of colonization in our experiments, we detected significant impacts of AMF on susceptibility of rice to both below- and aboveground pest organisms. We found that AMF inoculation caused a strong positive effect on the performance of the leaf-feeding insect FAW and the root-feeding RWW, as well as on the severity of disease caused by a fungal pathogen. The increased susceptibility of rice to herbivores and a pathogen in AMF-inoculated plants was not associated with changes in plant nutrient concentrations but was associated with an increase in shoot biomass. Taken together, these results show that the interactions of rice roots with AMF caused a broad-spectrum reduction in resistance to pests of rice, perhaps by altering defense-related pathways.

The increases in susceptibility to RWW in AMF-inoculated field plots, particularly in Exp-1, were greater than the differences in RWW densities typically observed among resistant and susceptible varieties of rice (N’Guessan et al., 1994; Stout et al., 2001), suggesting that the symbiotic status of rice plants might be a crucial component of susceptibility to RWW in the field. There was, however, some variability in the response of rice to AMF inoculation. In the second and third core samplings of Exp-2, and again in the second core sampling of Exp-3, densities of immature RWW did not differ between the M and NM treatments. The reasons for this variability in response to AMF inoculation are unknown. One possible reason is that sample and plot sizes might not have been sufficiently large to detect a weak effect of AMF inoculation among treatments, and it is interesting to note that all means in all core samplings trended in the direction of higher weevil densities in AMF-inoculated plants. Furthermore, experiments in 2012, when effects of AMF inoculation were large, and experiments in 2013, when effects were smaller, utilized different rice varieties (‘Lemont’ in 2012 and ‘Cocodrie’ in 2013), and were subject to different environmental conditions because they were conducted in different fields. With respect to the effect of rice variety, plant responses to AMF inoculation are known to vary among varieties within a plant species (Sawers et al., 2010).

The effectiveness of our experimental treatments in establishing AMF symbiosis was verified by quantifying AMF colonization in root samples in seven of our experiments. Although AMF colonization was not verified in all individual experiments, the substantial and statistically significant increases in colonization in response to commercial inoculants in the seven experiments in which colonization was assessed supports the postulation that addition of inoculum led to increased colonization in experiments in which mycorrhizal colonization was not quantified. An unresolved question in our experiments is whether actual colonization of rice roots differed among the six species of fungi in our inoculum, as we did not examine changes in colonization by individual fungal species. Different species and combinations of AMF are known to have different effects on plant resistance to herbivores (Gange, 2001; Roger et al., 2013).

The effects of AMF colonization on plant-herbivore and plant-pathogen interactions have been variable in previous studies (Gange, 2001; Bennett and Bever, 2007; Hartley and Gange, 2009; Koricheva et al., 2009; Currie et al., 2011; Jung et al., 2012; Barber et al., 2013a). The effects of AMF colonization on herbivores and pathogenic microorganisms depend on numerous factors, including host plant species, AMF species, herbivores or pathogens involved, and environmental conditions (Pineda et al., 2010). Our study contributes to a growing body of evidence that the effects of AMF in plants do not always lead to priming of plant tissues for a more efficient activation of defense mechanisms (Pozo and Azcon-Aguilar, 2007). This study also extends a previous report of positive effects of AMF inoculation on RWW oviposition (Cosme et al., 2011) and shows that the positive effects of AMF inoculation on RWW are observed in different developmental stages of RWW. Furthermore, the oviposition preference of RWW for mycorrhizal over nonmycorrhizal plants (Cosme et al., 2011) coupled with the higher performance of RWW larvae on mycorrhizal plants (this study) provides support for the preference-performance hypothesis for belowground herbivores, which predicts that when insect herbivores have offspring with limited mobility, there will be strong selection pressure for adults to oviposit on plants that maximize offspring performance (Johnson et al., 2006).

As noted above, several previous studies have, like this one, found positive effects of AMF inoculation on herbivore performance. Currie et al. (2011) found colonization of clover plants by AMF increased on survival of larvae of the specialist clover root weevil (*Sitona lepidus*). Likewise, Goverde et al. (2000) reported that survival and larval weights of the common blue butterfly (*Polyommatus icarus*) were greater in larvae that fed on *Lotus corniculatus* plants colonized by AMF. Gange et al. (2002) demonstrated that AMF colonization increased the larval growth of the specialists lace border (*Scopula ornata*), mint moth (*Pyrausta aurata*), and redcurrant aphid (*Cryptomyzus ribis*) on plants in the Lamiaceae family. The stronger performance of RWW, an oligophagous insect that specializes on grasses, on AMF-inoculated rice is consistent with results of a meta-analysis (Koricheva et al., 2009) that noted a general pattern in which most specialist chewing insects, but not most generalist insects, perform better on plants colonized by AMF than on non-colonized plants. However, our results with the generalist FAW, which showed higher larval growth on AMF-inoculated rice plants, contradicts this general pattern. Gange et al. (2002), similarly found that AMF colonization had a positive effect on the growth of the generalist aphid (*Myzus persicae*), and Hoffmann et al. (2009) showed that females of the generalist two-spotted spider mite (*Tetranychus urticae*) preferentially resided and oviposited at a higher rate on common bean plants colonized by AMF.

The effects of AMF colonization on aboveground pathogenic microorganisms have also been investigated in several prior studies. In rice in particular, Campos-Soriano et al. (2011) found that AMF confers enhanced rice resistance against infection by the rice blast fungus. In our experiments with ShB, we found that mycorrhizal rice plants were more susceptible to infection by *R. solani* than nonmycorrhizal plants. Because flooded rice plants were used in our study, and non-flooded plants in the study by Campos-Soriano et al. (2011), it is possible that water regime might affect the impact of AMF on rice resistance to ShB, although other experimental differences may also have contributed to these contrasting results. Altogether, our results underscore the variability of the effects of AMF colonization in plant-insect and plant-pathogen interactions.

There are three major hypotheses to explain the increases in rice susceptibility when colonized by AMF in this study. First, the interaction of AMF with rice might increase susceptibility to pests by increasing plant quantity (biomass) with no change in plant quality. Bennett et al. (2006) refer to this hypothesis as the “nutritional quantity hypothesis.” Second, AMF colonization might increase the quality of plant tissues for herbivores by improving plant nutrient status, which is referred by Bennett et al. (2006) as the “nutritional quality hypothesis.” In our experiments, we found no support for the nutritional quality hypothesis; no significant differences in concentrations of P, N, K and C, the nutrients that are most frequently studied in plant-AMF experiments, were found among AMF-inoculated plants and non-inoculated controls. In a previous study using the same rice-RWW system, however, Cosme et al. (2011) found that increased oviposition preference of RWW adults on mycorrhizal rice plants was associated with increased N and P concentrations. The effects of AMF on plant nutritional status have been widely studied in other systems, particularly effects of AMF on P, where P deficiency in soil promotes mycorrhizal formation (Secilia and Bagyaraj, 1994; Cosme and Wurst, 2013; Babikova et al., 2014b). In contrast to the results for nutrient status, we observed that AMF inoculation increased shoot biomass of rice plants in field and greenhouse studies (**Table 3**), which is in agreement with previous studies (Campos-Soriano et al., 2010). This result is consistent with the nutritional quantity hypothesis for RWW first instars, FAW and ShB, which live on aboveground plant tissues. However, the relatively moderate increases in shoot biomass observed are unlikely to fully account for the substantial increases in susceptibility to pests found in greenhouse experiments. This is particularly true for the increase in FAW susceptibility, as the FAW assay used excised leaf tissue and insects were never food-limited.

A third major hypothesis to explain increases in rice susceptibility in this study involves AMF-mediated changes in the expression of plant defenses via modulation of phytohormone signaling and consequent reprogramming of defense-related gene expression and other processes (Jung et al., 2012; Gutjahr, 2014; Pozo et al., 2015). There is evidence that AMF colonization can prime or otherwise affect jasmonic acid (JA)- and salicylic acid (SA)-dependent pathways (Pozo and Azcon-Aguilar, 2007; Herrera-Medina et al., 2008; Koricheva et al., 2009; Jung et al., 2012), and that these changes in plant signaling can lead to enhanced or decreased plant resistance against herbivores or pathogens (Campos-Soriano et al., 2011; Jung et al., 2012). Fontana et al. (2009) demonstrated that mycorrhizal symbiosis induced qualitative and quantitative changes in the production of volatile compounds of *Plantago lanceolata* plants when they were infested by caterpillars of *Spodoptera* spp. In another study, Jung et al. (2012) reported that AMF plants were usually more resistant to necrotrophs and chewing insects, which are affected by JA-dependent defense responses, and more susceptible to biotrophs (Jung et al., 2012). Thus, the evolution of plant-AMF interactions has apparently resulted in a repertoire of responses to AMF colonization that influence interactions with insects and pathogens (Gehring and Bennett, 2009; Gutjahr and Paszkowski, 2009; Kiers et al., 2010; Jung et al., 2012; Babikova et al., 2014a,b; Gilbert and Johnson, 2015; Pozo et al., 2015). However, the impact of AMF on plant defense hormone levels and gene transcription vary depending on the genotypes of the partners and other factors (Fernández et al., 2014).

In rice in particular, inoculation of unflooded roots with AMF induces a complex transcriptomic reprogramming, leading to enrichment of transcripts associated with phytohormones and secondary metabolism (Fiorilli et al., 2015; Gutjahr et al., 2015a). In our study, the fact that large effects of AMF inoculation on plant resistance were observed despite low levels of AMF colonization suggest that inoculation with AMF induced a systemic reprogramming of defense-related processes. However, the exact AMF-induced changes in JA and SA signaling and consequent changes in gene expression that influence the systemic susceptibility of wetland rice remain to be elucidated. Work is in progress to investigate expression levels of genes involved in the JA and SA signaling pathways of leaf tissues following AMF inoculation and FAW feeding using an RNA-seq and real time -PCR.

In summary, this study demonstrates that inoculation of rice plants with AMF rendered the plants more susceptible to pests without causing dramatic changes in plant nutrient concentrations. Our study highlights that AMF can compromise plant resistance and suggests that caution should be used when considering large scale applications of commercial AMF inoculant. However, despite the negative effects on plant resistance observed in this study, it would be premature to conclude that AMF does not have practical benefits for rice production. The higher shoot biomass of AMF-inoculated plants observed in two experiments in this study suggests that AMF inoculation may positively impact rice growth and perhaps yields under some circumstances. Moreover, the negative impact of AMF on plant resistance may not occur in all soil environments. Barber et al. (2013a), for example, found that the effects of AMF on plant nutrition vary with soil source and therefore soil characteristics may influence the effects of AMF colonization on herbivores. Although the effects of AMF on rice susceptibility were consistent in our study, the strength of these effects appeared to vary under the different conditions present in different experiments. Work is in progress to investigate whether different soil attributes, (e.g., soil P concentrations), alter the effects of AMF inoculation on the performance and growth of RWW and FAW in rice. Moreover, experiments are also being conducted to characterize the impacts of AMF inoculation on rice growth and yield when insects are not present. Responses to AMF provide a unique window for studying the traits or characteristics that make rice plants more susceptible or tolerant to insect and pathogen attack. A better understanding of the interactions of rice and other crops with AMF in the rhizosphere and with the different organisms they encounter both above and below ground may be a key to increasing plant productivity and improving pest management with less input of harmful chemicals.

## Author Contributions

LB, MC, and MS designed the experiments, which were then carried out by LB and MS. RS advised with the root staining technique, and LB improved it. LB collected the data and conducted the statistical analyses. LB, MC, and MS contributed to interpreting the results. LB wrote the first draft of the manuscript. All authors edited the manuscript and gave final approval for publication.

## Conflict of Interest Statement

The authors declare that the research was conducted in the absence of any commercial or financial relationships that could be construed as a potential conflict of interest.
