# Supplementary material for: Belowground Inoculation With Arbuscular Mycorrhizal Fungi Increases Local and Systemic Susceptibility of Rice Plants to Different Pest Organisms
Source: Front Plant Sci. 2018 Jun 5;9:747. doi: 10.3389/fpls.2018.00747 (PMC5996305; doi:10.3389/fpls.2018.00747)
Supplement: Supplementary file 1 [file Table_1.pdf]

Supplementary Material

Colonization by arbuscular mycorrhizal fungi increases susceptibility of rice, *Oryza sativa* L., to pest organisms

Lina Bernaola[1]*, Marco Cosme[2], Raymond Schneider[3], Michael Stout[1]

*** Correspondence:** Lina Bernaola: lbernaola@agcenter.lsu.edu

1 Supplementary Tables

Table S1. Properties of soil collected from the Crowley site for experiments conducted in 2012 and 2013

Soil Properties	2012		2013	
	**Amount**	**Rating***	**Amount**	**Rating***
Texture	Silt loam		silt loam	
pH (in water)	5.57	OP	5.97	OP
% organic matter	2.33		1.77	
CEC	9		12.54	
P (ppm)	11	L	13	L
K (ppm)	110	H	74	M
Ca (ppm)	1,341	M	1202	VH
Mg (ppm)	459	VH	254	VH
Na (ppm)	138	OP	54	VL
S (ppm)	11.6	L	4.23	L
Cu (ppm)	1.8	H	1.39	H
Zn (ppm)	4.3	H	8.1	H

*According to recommendation sheet: http://www.stpal.lsu.edu\recsheets\C-150.RTF
CEC = cation exchange capacity; OP = optimal; L = low; M = medium; VH = very high; VL = very low; H = high.

Table S2. Summary of field and greenhouse experiments conducted in 2012 and 2013.

Year	Trial	Treatments	Nº of reps/ treatment	Nº plants/ treatment	Root colonization assessed
2012	Experiment-1	F, NM & M	8	> 400	No
	FAW1	NM & M	14	42	No
2013	Experiment-2	F, NM & M	10	> 400	Yes
	Experiment-3	F, NM & M	10	> 400	Yes
	RWW1	NM & M	14	28	Yes
	RWW2	NM & M	12	36	Yes
	FAW2	NM & M	15	45	Yes
	FAW3	NM & M	15	45	Yes
	ShB1	NM & M	15	75	Yes
	ShB2	NM & M	15	45	No
	PB1	NM & M	12	48	No

The F, NM and M refer to AMF treatments of F: rice seeds + fungicides + sterilized AMF, NM: rice seeds + sterilized AMF, and M: rice seeds + live AMF. The Experiment-1, 2 and 3 are field experiments conducted against the rice water weevil. The RWW1, RWW2, FAW1, FAW2, FAW3, ShB1 and ShB2 experiments were conducted against the rice water weevil, fall armyworm and sheath blight of rice. The PB1 refers to plant biomass greenhouse experiment using field soil.

Table S3. Results of ANOVA (Proc Mixed) of arbuscular mycorrhizal fungi (AMF) treatment effects on plant (root and shoot tissue) nutrient concentration of 30-day-old rice plants taken from field and greenhouse experiments in 2012 and 2013.

**Root tissue**	**N (%)**	**P (%)**	**K (%)**	**C (%)**
**Field 2012 (Exp-1)**	Mean ± SE	Mean ± SE	Mean ± SE	Mean ± SE
Fungicide (F)	1.80 ± 0.08	0.12 ± 0.01	1.15 ± 0.02	37.08 ± 1.18
Nonmycorrhizal (NM)	1.71 ± 0.02	0.11 ± 0.01	1.11 ± 0.07	36.45 ± 1.18
Mycorrhizal (M)	1.86 ± 0.02	0.13 ± 0.01	1.19 ± 0.07	38.28 ± 0.19
$F_{2,6}$	3.59	3.59	0.95	0.91
P-value	0.095	0.094	0.437	0.451
**Field 2013 (Exp-2)**	Mean ± SE	Mean ± SE	Mean ± SE	Mean ± SE
Fungicide (F)	0.78 ± 0.03	0.13 ± 0.01	0.89 ± 0.07	35.55 ± 2.36
Nonmycorrhizal (NM)	0.89 ± 0.04	0.14 ± 0.01	1.06 ± 0.05	36.55 ± 1.93
Mycorrhizal (M)	0.87 ± 0.03	0.15 ± 0.01	0.98 ± 0.05	36.73 ± 0.83
$F_{2,6}$	2.67	0.68	2.09	0.12
P-value	0.148	0.543	0.204	0.889
**GH 2013 (PB1)**	Mean ± SE	Mean ± SE	Mean ± SE	Mean ± SE
Nonmycorrhizal (NM)	1.41 ± 0.05	0.19 ± 0.01	1.26 ± 0.06	39.23 ± 0.38
Mycorrhizal (M)	1.41 ± 0.06	0.18 ± 0.01	1.26 ± 0.04	38.75 ± 0.25
$F_{1,5}$	0.00	1.00	0.01	1.14
P-value	0.980	0.364	0.911	0.335

The F, NM and M refer to AMF treatments of F: rice seeds + fungicides + sterilized AMF, NM: rice seeds + sterilized AMF, and M: rice seeds + live AMF. Concentrations of four elements did not differ significantly among treatments.

(Continue next page)

Shoot tissue	N (%)	P (%)	K (%)	C (%)
**Field 2012 (Exp-1)**	Mean ± SE	Mean ± SE	Mean ± SE	Mean ± SE
Fungicide (F)	3.15 ± 0.04	0.16 ± 0.01	2.56 ± 0.06	40.45 ± 0.26
Nonmycorrhizal (NM)	3.05 ± 0.14	0.14 ± 0.01	2.44 ± 0.07	39.75 ± 0.10
Mycorrhizal (M)	3.25 ± 0.05	0.16 ± 0.01	2.45 ± 0.03	40.28 ± 0.24
$F_{2,6}$	2.01	0.65	1.62	3.80
P-value	0.214	0.554	0.275	0.086
**Field 2013 (Exp-2)**	Mean ± SE	Mean ± SE	Mean ± SE	Mean ± SE
Fungicide (F)	1.45 ± 0.12	0.27 ± 0.01	2.09 ± 0.12	39.48 ± 0.32
Nonmycorrhizal (NM)	1.70 ± 0.09	0.29 ± 0.01	2.19 ± 0.12	39.85 ± 0.17
Mycorrhizal (M)	1.45 ± 0.12	0.29 ± 0.01	2.09 ± 0.04	39.45 ± 0.21
$F_{2,6}$	3.50	2.75	0.73	0.87
P-value	0.098	0.142	0.518	0.467
**GH 2013 (PB1)**	Mean ± SE	Mean ± SE	Mean ± SE	Mean ± SE
Nonmycorrhizal (NM)	1.95 ± 0.06	0.23 ± 0.01	3.42 ± 0.05	38.11 ± 0.09
Mycorrhizal (M)	2.13 ± 0.13	0.22 ± 0.01	3.28 ± 0.05	38.18 ± 0.13
$F_{1,11}$	2.26	1.54	3.89	0.18
P-value	0.161	0.241	0.074	0.679

The F, NM and M refer to AMF treatments of F: rice seeds + fungicides + sterilized AMF, NM: rice seeds + sterilized AMF, and M: rice seeds + live AMF. Concentrations of four elements did not differ significantly among treatments.

2 Supplementary Figures

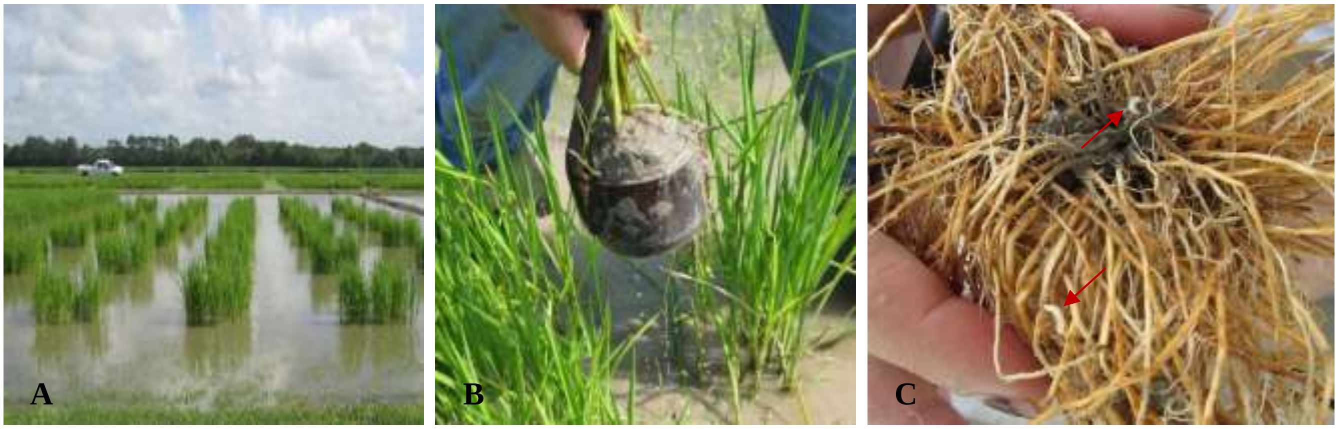

Figure S1. Photographic representation of rice water weevil injury. A: rice field under flooded conditions triggers rice water weevil infestations; B: core sampler used to collect plants from rice plots to determine weevil densities; and C: red arrows pointing larvae of rice water weevil feeding in rice roots.

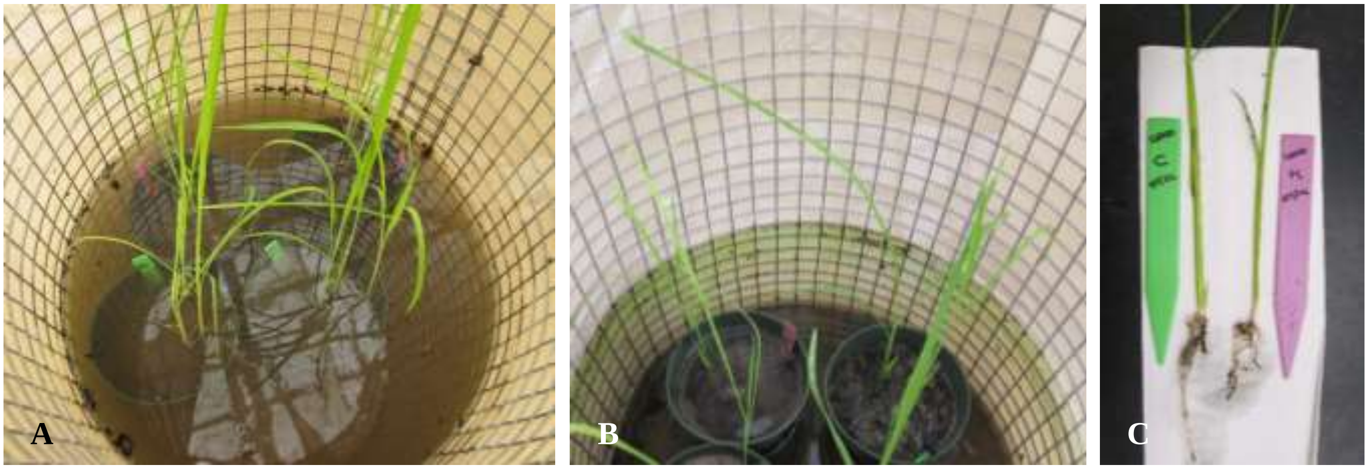

Figure S2. Photographic representation of rice water weevil choice experiments. A: mycorrhizal and nonmycorrhizal pots placed in a cage under flooded conditions before weevil infestation; B: mycorrhizal and nonmycorrhizal pots showing leaf injury (white scars) after weevil infestation; and C: mycorrhizal and nonmycorrhizal plants showing differences in the root system after weevil feeding.

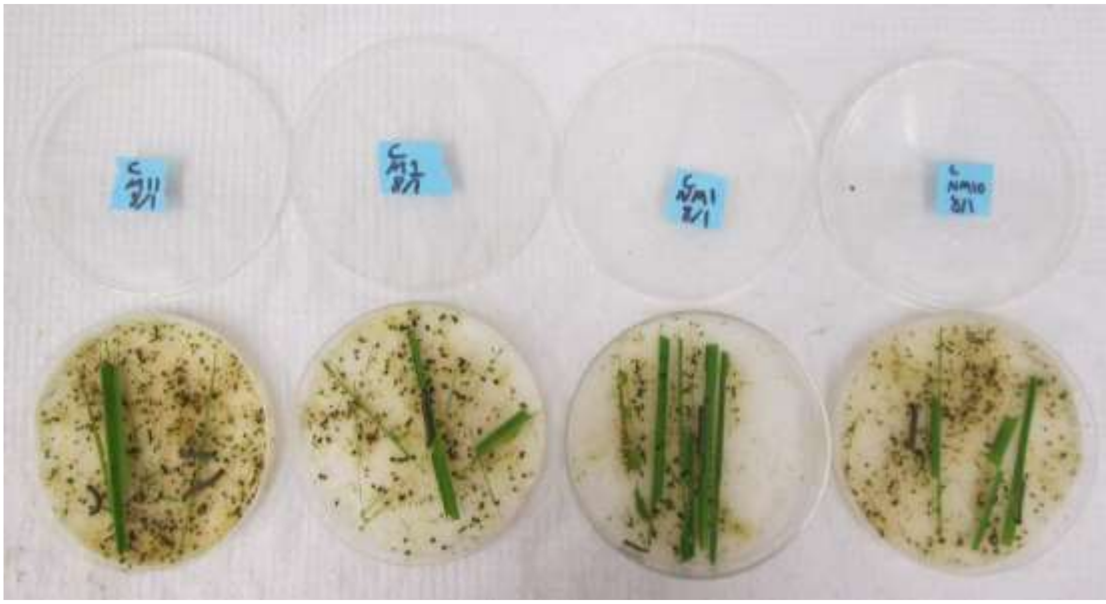

Figure S3. Photographic representation of typical fall armyworm feeding assays. Feeding assays were conducted in petri dishes lined with moistened cotton batting to maintain turgor in freshly cut leaf tissues. This picture shows difference among treatments (mycorrhizal and nonmycorrhizal tissues) at the end of the fall armyworm feeding experiment.

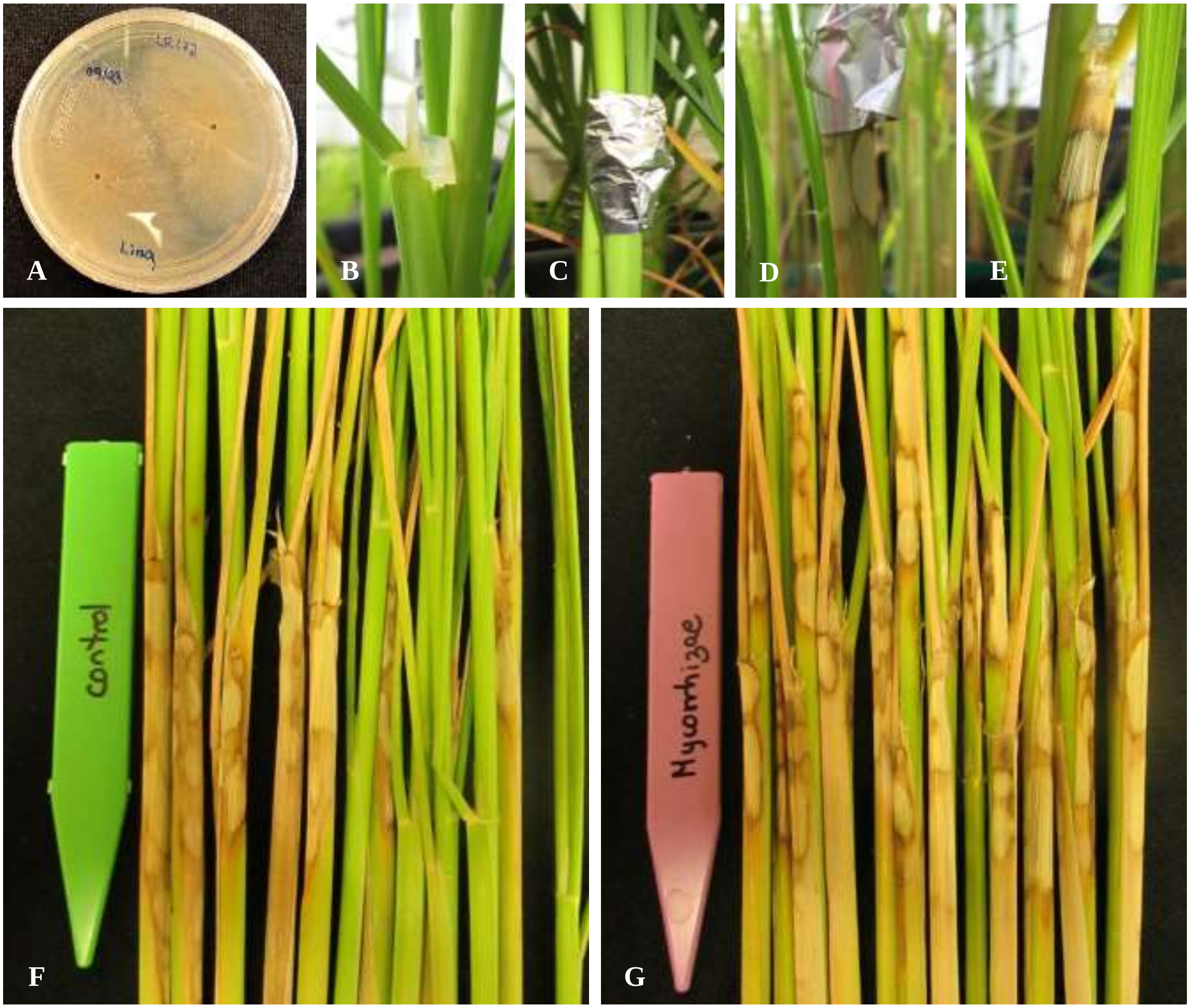

Figure S4. Photographic representation of sheath blight inoculation. A: sclerotia of *Rhizoctonia solani* on potato dextrose agar; B: inoculation of mycelia ball beneath leaf sheath; C: inoculated sheath covered with aluminum foil; D: appearance of lesions (symptoms) 3 days after inoculation; E: removal of aluminum foil 7 days after inoculation; F and G: level of infection in nonmycorrhizal and mycorrhizal rice plants, respectively.

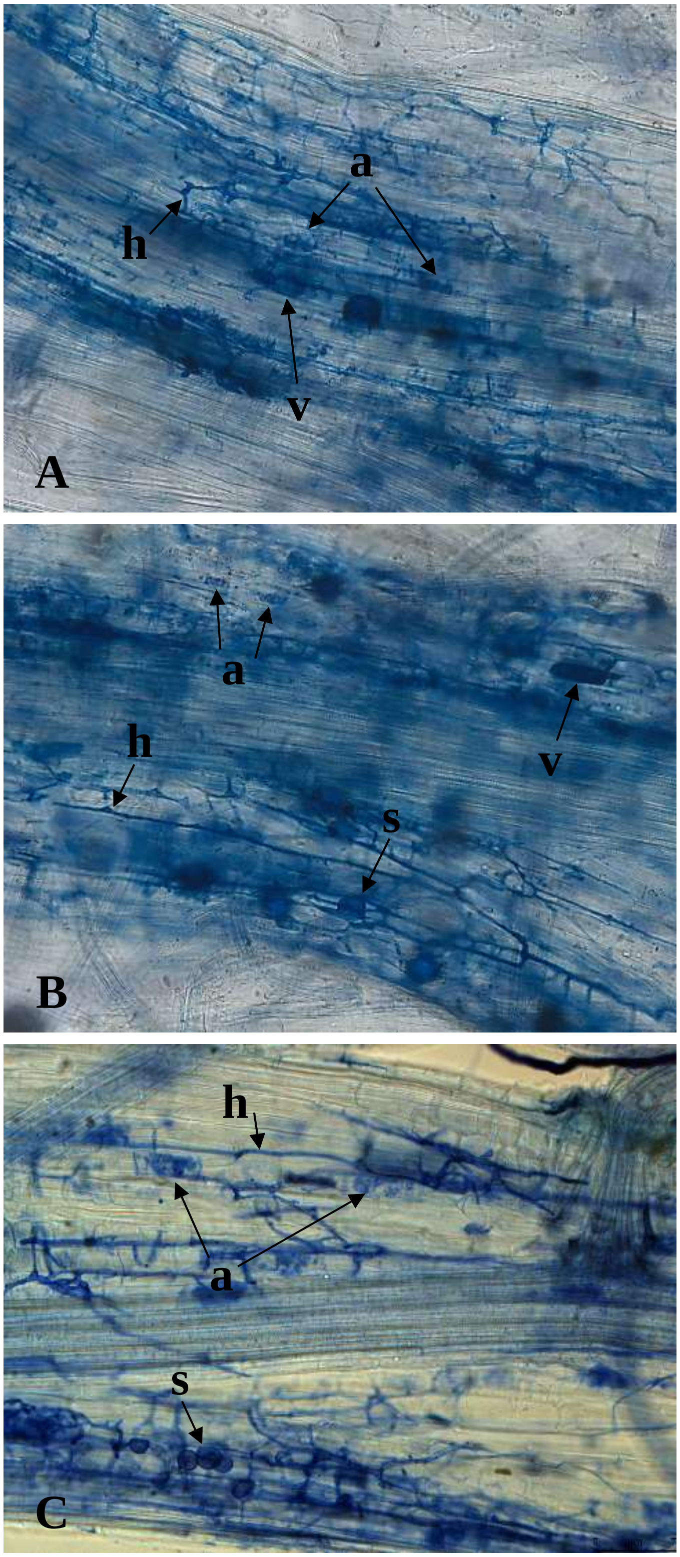

Figure S5. Root fragments stained with trypan blue showing arbuscular mycorrhizal fungi structures in rice plants. Light micrographs of mycorrhizal inoculated root fragments from some experiments

conducted in 2013 show: (A) Hyphae (h), arbuscule (a), and vesicle (v). (B) Hyphae, arbuscule, spore (s) and vesicle. (C) Hyphae, arbuscule, and spore.
